# Synergistic Inhibition of Nav1.7 and NCX1: A Novel Strategy for Treating Cancer‐Induced Bone Pain by Modulating Pain Sensitization and Neuronal Inflammation

**DOI:** 10.1111/cns.70389

**Published:** 2025-04-18

**Authors:** Yan Feng, Fang Yan, Dongtai Chen, Peizong Wang, Yan Yan, Xiangnan Chen, Qiang Li, Wei Xing, Weian Zeng, Yang Huang

**Affiliations:** ^1^ Department of Anesthesiology, State Key Laboratory of Oncology in South China, Guangdong Provincial Clinical Research Center for Cancer Sun Yat‐Sen University Cancer Center Guangzhou China; ^2^ Department of Anesthesiology Huizhou Municipal Central Hospital Huizhou China; ^3^ Department of Anesthesiology Guangdong Women and Children Hospital Guangzhou China

**Keywords:** cancer‐induced bone pain, neuroinflammatory response, pain sensitization, sodium‐calcium exchanger 1 (NCX1), synergistic analgesia, voltage‐gated sodium channel 1.7 (Nav1.7)

## Abstract

**Aims:**

Cancer‐induced bone pain (CIBP) is a chronic and refractory pain condition characterized by neuronal hyperexcitability, calcium dysregulation, and neuroinflammation. Voltage‐gated sodium channels (VGSCs) and sodium/calcium exchangers (NCXs) are crucial in regulating sensory neuron sodium–calcium homeostasis, influencing nociceptive signaling and neuroinflammatory responses. This study focused on exploring how Nav1.7 from the VGSC family and NCX1 from the NCX family influence nociceptive signaling and neuroinflammation in CIBP.

**Methods:**

CIBP was induced in mice. Nav1.7 and NCX1 expression and colocalization in DRG neurons were analyzed by qPCR, western blotting, and immunofluorescence. Calcium overload and neuronal excitability were assessed using calcium imaging and electrophysiological recordings. Neuroinflammation markers were detected by qPCR and western blotting.

**Results:**

Among the VGSC and NCX subtypes, Nav1.7 and NCX1 were notably upregulated and colocalized in the DRG neurons of CIBP mice. Combined inhibition of these channels demonstrated a synergistic analgesic effect and markedly reduced neuronal calcium overload and hyperexcitability. Furthermore, the combined inhibition substantially alleviated neuroinflammation by inhibiting the p38 MAPK/NF‐κB pathway and lowering proinflammatory cytokine levels.

**Conclusions:**

The combined inhibition of Nav1.7 and NCX1 enhances analgesic effects and reduces neuroinflammation, presenting a potential therapeutic approach for CIBP and other cancer‐associated pain disorders.

## Graphical abstract

Nav1.7‐mediated sodium influx promotes NCX1 reverse transport, increasing sensory neuron excitability and causing calcium overload, which activates the p38 MAPK/NF‐κB pathway and induces neuroinflammation. Dual inhibition reduces hyperexcitability, restores calcium homeostasis, suppresses neuroinflammation, and ultimately alleviates cancer‐induced bone pain (CIBP).

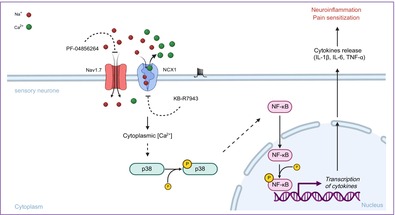

## 1 Introduction

Cancer‐induced bone pain (CIBP) is a complex and often intractable pain condition commonly observed in individuals with late‐stage cancer, particularly those suffering from bone metastases originating from breast, prostate, or lung cancer [1, 2, 3]. CIBP includes features of neuropathic, traumatic, and inflammatory pain, with underlying mechanisms that include nerve injury, neuroinflammation, altered excitability of peripheral neurons, and neuroplastic changes [4, 5, 6]. Patients with CIBP typically experience persistent background pain, accompanied by intermittent episodes of severe breakthrough pain [5, 7]. However, current analgesic therapies often provide limited relief and are associated with significant adverse effects.

Despite the availability of opioids, NSAIDs, and multimodal therapies such as radiotherapy and bisphosphonates, pain control remains inadequate. Opioids, though effective, are associated with tolerance, dependence, sedation, nausea, and constipation [8, 9, 10], while prolonged NSAID use increases the risk of gastrointestinal hemorrhage and cardiovascular complications [11, 12, 13]. Moreover, existing multimodal approaches, including radiotherapy, bisphosphonates, and tumor ablation, provide limited relief, particularly for activity‐related or breakthrough pain [14, 15, 16, 17]. These limitations underscore the need for alternative therapeutic strategies targeting novel molecular mechanisms.

Recent studies have highlighted the essential function of ion channels in pain signaling and neuroinflammation in CIBP, positioning them as promising therapeutic targets [4, 18, 19]. Voltage‐gated sodium channels (VGSCs) and sodium–calcium exchangers (NCXs) are key regulators of intracellular sodium–calcium homeostasis and are essential for nociception and neuroinflammation. Nav1.7, a member of the VGSC family, is primarily expressed in peripheral sensory neurons and is crucial for pain perception. Gain‐of‐function mutations in Nav1.7 are associated with familial erythromelalgia [20], small‐fiber neuropathy [21], and diabetic neuropathic pain [22], while inhibition of Nav1.7 function helps alleviate neuropathic and inflammatory pain [23, 24, 25]. Notably, Nav1.7 is more abundantly expressed in human VGSCs than in rodent models, indicating its greater relevance in human pain perception [26].

Similarly, NCX1, the predominant sodium‐calcium exchanger isoform in sensory neurons, plays a critical role in calcium homeostasis and has been implicated in neurological disorders such as Alzheimer's disease [27], amyotrophic lateral sclerosis [28], and hypoxic–ischemic encephalopathy [29]. Inhibiting NCX reverse‐mode transport reduces abnormal calcium accumulation and mitigates pain hypersensitivity following nerve injury [30].

Interestingly, studies have revealed a functional interaction between VGSCs and NCX in Alzheimer's disease and astrocytic proliferation following central nervous system injury, where they jointly regulate sodium‐calcium homeostasis and influence neuroinflammation and cellular proliferation [31, 32]. These findings indicate that VGSC–NCX interactions may serve as a fundamental regulatory mechanism across multiple disease states. However, whether Nav1.7 and NCX1 functionally interact in CIBP and contribute to pain sensitization and neuroinflammation remains unexplored.

To fill this knowledge gap, we sought to explore the functional role of Nav1.7 and NCX1 in CIBP and evaluate whether their combined inhibition can produce synergistic analgesic and anti‐neuroinflammatory effects. By elucidating the interaction between Nav1.7 and NCX1 in CIBP, this study aims to assess their potential as multitarget therapeutic strategies and provide new insights for the management of CIBP.

## 2 Materials and Methods

### 2.1 Experimental Animals and Model Establishment

#### 2.1.1 Animals

Male C57BL/6 mice (5–6 weeks old, 18–22 g) were obtained from the Guangdong Medical Laboratory Animal Centre (Guangzhou, China) and housed in a controlled environment with a 12‐h light/dark cycle, a temperature of 22°C ± 2°C, and humidity ranging from 50% to 60%, with unrestricted access to water and chow. To minimize variability, only males were used, as hormonal differences may affect pain sensitivity and inflammation. All procedures adhered to the NIH Guide for the Care and Use of Laboratory Animals and were approved by the Animal Care and Use Committee of Sun Yat‐sen University Cancer Centre (approval no. L025501202302009).

#### 2.1.2 Cancer‐Induced Bone Pain (CIBP) Model

Following established protocols, the CIBP model was developed [33]. The mice were anesthetized using isoflurane (2% for induction, 1.5% for maintenance), and a 0.5 cm incision was created above the left knee to reveal the patellar ligament. The ligament was moved laterally to expose the femoral condyle. A 25‐gauge insulin needle created a hole in the intercondylar notch of the femur. Lewis Lung Carcinoma‐luciferase (LLC‐luc) cells (3 × 10^5^ in 10 μL PBS) were injected into the medullary cavity using a Hamilton syringe, followed by 2 μL of Matrigel (Corning, Cat. No. 365234) to prevent leakage and promote cell growth. The incision was then sutured. Sham‐operated mice underwent an identical procedure but were injected with heat‐inactivated cells.

#### 2.1.3 In Vivo Bioluminescence and 3D CT Imaging

Bioluminescence imaging was performed weekly starting from 7 days post‐surgery to track tumor growth and progression. Before imaging, mice received an intraperitoneal dose of D‐luciferin potassium salt (150 mg/kg, dissolved in PBS: Solarbio, Cat. No. D8390) and were rested for 15 min. Under 2% isoflurane anesthesia, the mice were positioned in the IVIS Spectrum imaging system, and images were captured via a 520 nm longpass emission filter with a 5 s exposure.

Simultaneously, 3D CT imaging was performed weekly, beginning on day 7 post‐surgery, to assess the bone structure and lesion development. Scans were carried out under isoflurane anesthesia via a Quantum GX2 micro‐CT scanner (PerkinElmer, Waltham, MA, USA) at 70 kV with a 14‐min scan duration.

#### 2.1.4 Hematoxylin and Eosin (H&E) Staining

On day 21 post‐implantation of LLC‐luc cells, femurs from the tumor‐bearing side (left) were harvested and preserved in 4% paraformaldehyde at 4°C for 24 h. Decalcification was performed in a 10% EDTA solution (pH 7.4) at ambient temperature for 2–3 weeks before paraffin embedding. Tissue sections (7 μm thick) were mounted on glass slides and stained using the Solarbio HE Staining Kit (Cat. No. G1120) following the manufacturer's instructions. The stained samples were analyzed via a Nikon Eclipse Ni‐U microscope (Nikon, USA) to evaluate tumor growth and bone marrow structural changes.

### 2.2 Behavioral Testing

Behavioral assessments were conducted to evaluate pain‐related behaviors in both the CIBP model and control groups. Before baseline measurements, the mice gradually acclimated to the testing environment over several days to minimize stress‐related variability. Group allocation, drug administration, and behavioral testing were conducted in a blinded manner. All tests, including assessments of mechanical allodynia, thermal hyperalgesia, and hind limb use, were performed by blinded experimenters between 9:00 AM and 5:00 PM, with specific time points determined on the basis of experimental objectives.

#### 2.2.1 Von Frey Test

Mechanical allodynia, defined as a pain response to normally non‐painful stimuli, was assessed using the von Frey filament test. Mice were individually placed in transparent containers, allowing exposure of the plantar surface of their hind paws. Following a 30‐min acclimation period, von Frey filaments (0.02–4 g) were applied perpendicularly to the plantar surface for 1–2 s each. Each filament was applied five times, with a 30‐s interval between applications. A withdrawal, flick, or lick response in at least three out of five applications was recorded as a positive response, and the corresponding filament weight was defined as the mechanical withdrawal threshold (MWT).

#### 2.2.2 Hot Plate Test

Thermal hyperalgesia, characterized by increased sensitivity to heat, was evaluated using the hot plate test. Mice were positioned on a ZH‐6C hot plate set to 52.0°C ± 0.5°C to assess heat sensitivity. Following a 30‐min acclimation period, the latency to the first nociceptive response, such as paw licking or jumping, was recorded and defined as the thermal withdrawal latency (TWL). Each mouse was exposed to three heat stimuli, with a 5‐min rest between trials, and the average TWL was calculated. A 30 s cutoff was applied to avoid tissue damage.

#### 2.2.3 Hind Limb Use Scoring

The use of hind limbs was assessed to evaluate movement‐related pain in CIBP mice. The mice were placed in an open space for 3 min, and their spontaneous activity was observed. The scoring criteria were as follows: 4 points for normal limb use without discomfort; 3 points for mild impairment with slight reluctance or occasional limping; 2 points for moderate impairment with noticeable limping and asymmetric weight‐bearing; 1 point for severe impairment with minimal limb use and frequent signs of pain; and 0 points for complete loss of limb use or movement avoidance due to pain [34]. The mice were evaluated three times at various time points, with the mean score applied for data analysis.

### 2.3 Drug Treatment and Synergistic Analysis

#### 2.3.1 Drug Treatment

Veratridine, KB‐R7943, and PF‐04856264 were dissolved in dimethyl sulfoxide (DMSO) to achieve a 10 mM concentration and then aliquoted and kept at −80°C for later use.

For intrathecal injections in mice, stock solutions were gradually diluted in a solvent mixture of 40% PEG300, 5% Tween‐80, and saline, following the manufacturer's guidelines (MCE) to prepare the working solutions. This solvent system ensured complete dissolution and uniform dispersion of the drugs. The vehicle control group received the same solvent mixture without the active compounds. For in vitro experiments, the drugs were directly diluted to the appropriate concentrations in PBS or culture medium.

#### 2.3.2 Dose–Response Curve and Combination Index Calculations

This study used a four‐parameter logistic regression model to fit the dose–response curves of drugs and calculate the half–maximal effective dose (ED_50_) and related parameters. The drug effect (*Y*) at different dose levels (*x*) can be estimated via the equation below:

$$Y=\text{Bottom}+\frac{\mathrm{Top}-\text{Bottom}}{1+{\left(\frac{x}{{\mathrm{EC}}_{50}}\right)}^{-\text{Hill Slope}}}$$

In this equation, *Y* indicates the percentage of the effect, *x* represents the drug dose, and bottom and top correspond to the lowest and highest effect values, respectively. EC_50_ is the ED_50_, and the Hill slope represents the slope of the curve.

To evaluate the interaction of two drugs in combination, the combination index (CI) was calculated via the Chou–Talalay method. The CI was calculated as follows:

$$\mathrm{CI}=\frac{\frac{d_{A}}{D_{A}}+\frac{d_{B}}{D_{B}}}{\frac{E}{1-E}}$$

where *d*_A_ and *d*_B_ are the actual doses of drugs A and B in combination, respectively; *D*_A_ and *D*_B_ are the doses of drugs A and B required to achieve the same effect (*E*) when used individually; and *E* is the observed effect of the drug combination (expressed as a percentage). The doses of single drugs required to achieve specific effects were interpolated from their respective dose–response curves fitted with the logistic regression model, and the interpolation was performed via the fsolve function in SciPy. The CI is categorized as follows: synergism (CI < 1), additive effect (CI = 1), and antagonism (CI > 1). All data analysis and calculations were carried out in the Python environment (version 3.10.12).

The effectiveness rate (*E*) in this study was defined as an increase of at least one level in the mechanical pain threshold in the Von Frey test and a 30% or greater extension in the reaction latency in the hot plate test. The effectiveness rate was calculated as the percentage of data points meeting these criteria out of the total number of tests.

### 2.4 Calcium Imaging and Patch Clamp Experiments

#### 2.4.1 Isolation and Culture of DRG Neurons

Primary DRG neurons were isolated from 1‐week‐old C57BL/6 neonatal mice via a previously described protocol [35]. Collagenase (1 mg/mL) and trypsin (0.25%) were used to enzymatically digest the DRG tissues at 37°C for 30 min. The dissociated cells were then seeded onto 24‐well plates precoated with 0.01% (w/v) rat tail collagen. To increase neuronal purity, the adhered cells were treated with complete medium containing 10 μM cytosine arabinoside (Ara‐C) for 48 h to suppress glial cell growth. The complete medium included neurobasal medium, 2% B27, 0.5 mM glutamine, and 100 U/mL penicillin–streptomycin. The medium was refreshed with complete medium after 48 h, and the cultures were maintained for subsequent experiments.

#### 2.4.2 Calcium Imaging

DRG neurons were preincubated with PF‐04856264 (2 μM), KB‐R7943 (5 μM), or their combination for 30 min prior to calcium imaging. Cells were then loaded with the calcium‐sensitive dye Fluo‐4 AM (Beyotime, Cat# S1061S) in HEPES‐buffered saline (HBS) at 37°C for 30 min. After washing, neurons were maintained in HBS containing the respective inhibitors to ensure continuous inhibition.

Baseline fluorescence intensity (*F*_0_) was recorded for 25 s before stimulation with 30 μM veratridine, a voltage‐gated sodium channel activator. Fluorescence images were captured every 5 s using an LSM980 confocal microscope (Zeiss, Germany). Fluorescence changes were normalized to baseline values and expressed as Δ*F*/*F*_0_. The area under the curve (AUC) was calculated for each treatment group, representing the cumulative fluorescence response over the entire experimental period, which reflects the overall treatment effect. Data analysis was performed using GraphPad Prism.

#### 2.4.3 Patch Clamp Recording

Whole‐cell patch clamp recordings were obtained to measure neuronal action potentials. A high‐resistance seal (GΩ range) was created under negative pressure as recording electrodes (3–5 MΩ) filled with intracellular solution were placed on the cell surface. After the membrane rupture, a whole‐cell configuration was established with capacitance and series resistance adjustments. In current clamp mode, stimulus currents ranging from −50 to ~530 pA were applied in 20 pA increments (pulse duration: 0.8 s, interval: 1 s). Action potentials were recorded at 1.5–2 times the rheobase current for 1 s. Signals were acquired via an EPC 10 amplifier and stored in Patch Master software (HEKA). After stabilization of action potentials, drugs were sequentially applied via a gravity‐fed perfusion system, with extracellular solution‐treated cells serving as controls. The analyzed parameters included the spike number, maximum upstroke slope, and threshold potential. All experiments were conducted at ambient temperature.

### 2.5 Molecular and Cellular Experiments

#### 2.5.1 RNA Extraction and Quantitative RT–PCR

Total RNA was isolated from the left L4–L6 DRGs of tumor‐bearing and sham‐operated mice using a commercial RNA purification kit. cDNA was synthesized via reverse transcription, followed by qPCR on a CFX96 system (Bio‐Rad) using SYBR Green. Gene expression was calculated by the $2^{-\Delta \Delta C_{t}}$ method with GAPDH as the internal reference. Primer sequences are listed in Table S2.

#### 2.5.2 Western Blot Analysis

Protein was isolated from DRG tissues or cultured cells using RIPA buffer supplemented with protease and phosphatase inhibitors. Its concentration was determined via BCA assay. Samples underwent SDS‐PAGE separation, followed by transfer to PVDF membranes, blocking with 5% BSA, and overnight incubation with primary antibodies (Table S1). The next day, HRP‐conjugated secondary antibodies (ZenBio, China, Cat# 511203) were applied. Protein bands were visualized using enhanced chemiluminescence (ECL) and captured with a Bio‐Rad ChemiDoc system. Protein quantification was conducted via ImageJ, with GAPDH serving as a normalization control.

#### 2.5.3 Immunofluorescence Staining

Paraffin‐embedded L4–L6 DRG tissue sections were processed following fixation in 4% paraformaldehyde, dehydration, and antigen retrieval by heating in citrate buffer (pH 6.0) at 95°C. Blocking was performed with 5% goat serum for 1 h, after which the sections were incubated overnight at 4°C with primary antibodies (Table S1). The next day, Cy3‐conjugated goat anti‐rabbit IgG (1:1000, Jackson ImmunoResearch) and Alexa Fluor 488 goat anti‐mouse IgM μ chain (1:200, Abcam 150121) were incubated at room temperature for 1 h. DAPI was used for nuclear staining, and fluorescence images were acquired via an LSM980 confocal microscope (Zeiss, Germany).

Nav1.7 and NCX1 colocalization was evaluated via plot profile analysis via ImageJ software and Pearson's correlation coefficient, which ranges from −1 (no correlation) to 1 (complete colocalization). The coefficient was calculated from three independent images.

#### 2.5.4 shRNA Transfection in ND7/23 Cells

ND7/23 cells were grown in DMEM containing 15% FBS, 1% penicillin–streptomycin, and 2 mM glutamine at 37°C with 5% CO_2_. shRNA constructs targeting Nav1.7, NCX1, or both were transfected into cells at 70%–90% confluency via the Lip3000 transfection reagent (Huayun, China). The shRNA plasmids (single or combined) and Lip3000 reagent were separately diluted in Opti‐MEM (Gibco, USA) and mixed per the manufacturer's instructions to form complexes, which were introduced into the cells. Transfection was carried out over 48 h.

The knockdown efficiency was validated through western blotting to assess protein expression levels. The control cells were transfected with nontargeting shRNA. All the experiments were conducted in triplicate to ensure reproducibility.

### 2.6 Statistical Analysis

All data were presented as the mean ± SEM. The normality of the data distribution was assessed using the Shapiro–Wilk test. For comparisons between two groups, paired or unpaired Student's *t*‐tests were performed depending on the data structure. For comparisons involving multiple groups, one‐way or two‐way ANOVA followed by Bonferroni post hoc tests was used when the data met normality assumptions and homogeneity of variance. When these assumptions were not met, the Kruskal–Wallis test followed by Dunn's multiple comparisons test was applied. Dose–response curves were fitted via the SciPy curve fit function (v1.11.3, Python) with nonlinear least squares optimization, and ED50 confidence intervals (CIs) were calculated on the basis of the covariance matrix and Student's *t* distribution. The fitted parameters are reported as the means ± SEM, and the results were visualized with Matplotlib (v3.7.1). A significance threshold of *p* < 0.05 was applied, and all analyses were conducted in a blinded manner. Replicate details (*n*), statistical methods, and error measures are provided in the figure legends, with at least *n* = 3 biological replicates per experiment.

## 3 Results

### 3.1 Tumor‐Bearing Mice Exhibit Mechanical and Thermal Hyperalgesia, Significant Bone Destruction, and Severe Motor Impairment

**FIGURE 1:**
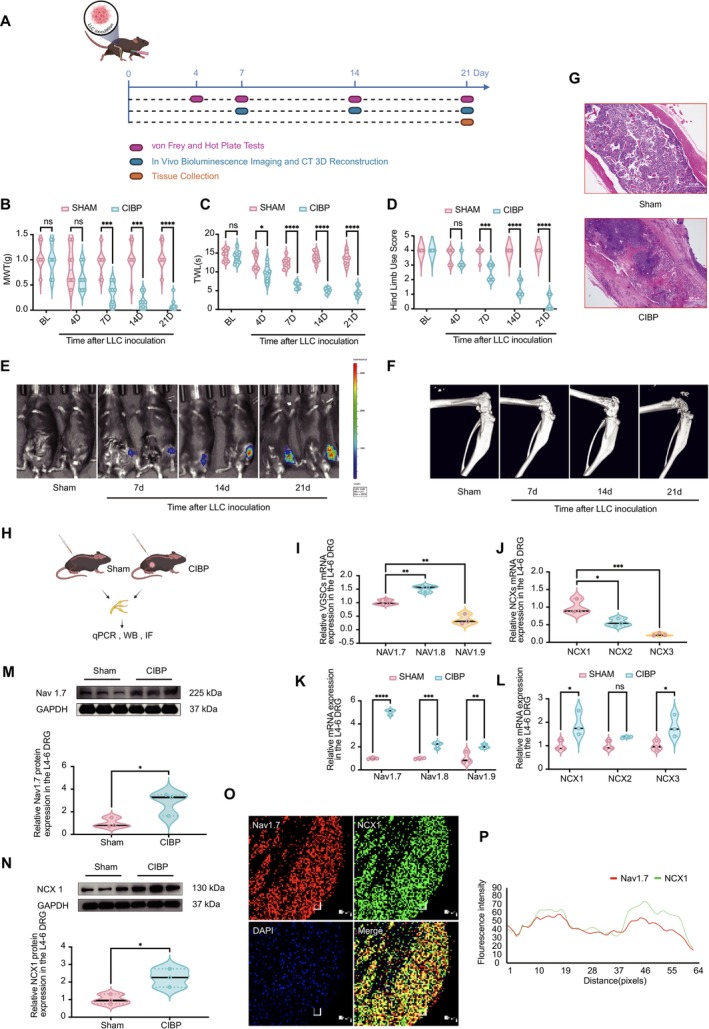
Establishment of the CIBP model and enhanced expression and colocalization of Nav1.7 and NCX1 in CIBP. (A) The experimental timeline illustrates the establishment of the CIBP model, behavioral tests (von Frey and hot plate), imaging evaluations (bioluminescence imaging and 3D CT scans), and femoral tissue collection for H&E staining at specific time points. (B–D) Behavioral assessments revealed MWT, TWL, and Hind limb use score in the CIBP group compared to the sham group. (E) Bioluminescence imaging indicated tumor progression in CIBP mice, while (F) CT scans demonstrated bone erosion. (G) H&E staining further confirmed bone destruction in CIBP mice. (H) L4–6 DRG tissues were collected on day 21 for qPCR, Western blot, and immunofluorescence analyses. (I, J) mRNA expression levels of VGSC and NCX family members in sham mice. (K, L) Relative expression levels of VGSC and NCX family members in CIBP mice compared to sham mice. (M, N) Western blot analysis confirmed significantly increased protein levels of Nav1.7 and NCX1 in the DRGs of CIBP mice. (O) Immunofluorescence staining and (P) fluorescence intensity analysis demonstrated a high degree of colocalization between Nav1.7 and NCX1 in DRG neurons. All data were tested for normality using the Shapiro–Wilk test and are presented as mean ± SEM. Statistical tests: (B, C, K, L) Two‐way ANOVA with Bonferroni post hoc test; (D) Kruskal–Wallis test with Dunn's post hoc test; (I, J) One‐way ANOVA with Bonferroni post hoc test; (M, N) Unpaired Student's *t*‐test. *p < 0.05, **p < 0.01, ***p < 0.001, ****p < 0.0001. *n* = 8 per group for (B–D); *n* = 3 per group for (I–O). CIBP, cancer‐induced bone pain; CT, computed tomography; DRG, dorsal root ganglia; H&E, hematoxylin and eosin staining; LLC, Lewis lung carcinoma; MWT, mechanical withdrawal threshold; NCX, sodium‐calcium exchanger; TWL, thermal withdrawal latency; VGSC, voltage‐gated sodium channel.

We established a bone metastasis cancer‐induced bone pain (CIBP) model by implanting Lewis lung carcinoma‐luciferase (LLC‐luc) cells into the femoral intramedullary cavity of C57BL/6 mice. To assess pain sensitivity and pain‐related motor function, behavioral tests were conducted at baseline (BL) and on days 4, 7, 14, and 21 post‐modeling. Tumor progression and bone destruction were evaluated through imaging analyses on days 7, 14, and 21, while histological changes in bone architecture were assessed via H&E staining on day 21 (Figure 1A). The MWT and thermal withdrawal latency TWL significantly declined from day 4 to 7 post‐implantation onwards. By day 21, MWT had decreased by 89.7% compared to the sham group (****p < 0.0001), while TWL had decreased by 65.5% (****p < 0.0001). Similarly, the hind limb use score was significantly reduced on day 21 compared to the sham group (****p < 0.0001) (Figure 1B–D). These findings indicate that bone metastasis induces pain sensitization and exacerbates movement‐related pain. Representative imaging figures visually demonstrate a trend similar to that observed in behavioral assessments. Bioluminescence imaging showed a sustained increase in tumor burden on days 14 and 21 (Figure 1E), while 3D micro‐CT imaging revealed progressive bone destruction, with early cortical erosion on day 7 and severe damage by day 21 (Figure 1F). Histopathological analysis confirmed extensive tumor cell infiltration and severe bone structural damage in CIBP mice, whereas the sham group maintained intact bone architecture (Figure 1G). Collectively, these findings confirm the successful establishment of the CIBP model.

### 3.2 Upregulation and Colocalization of Nav1.7 and NCX1 in the DRGs of CIBP Mice

**FIGURE 2:**
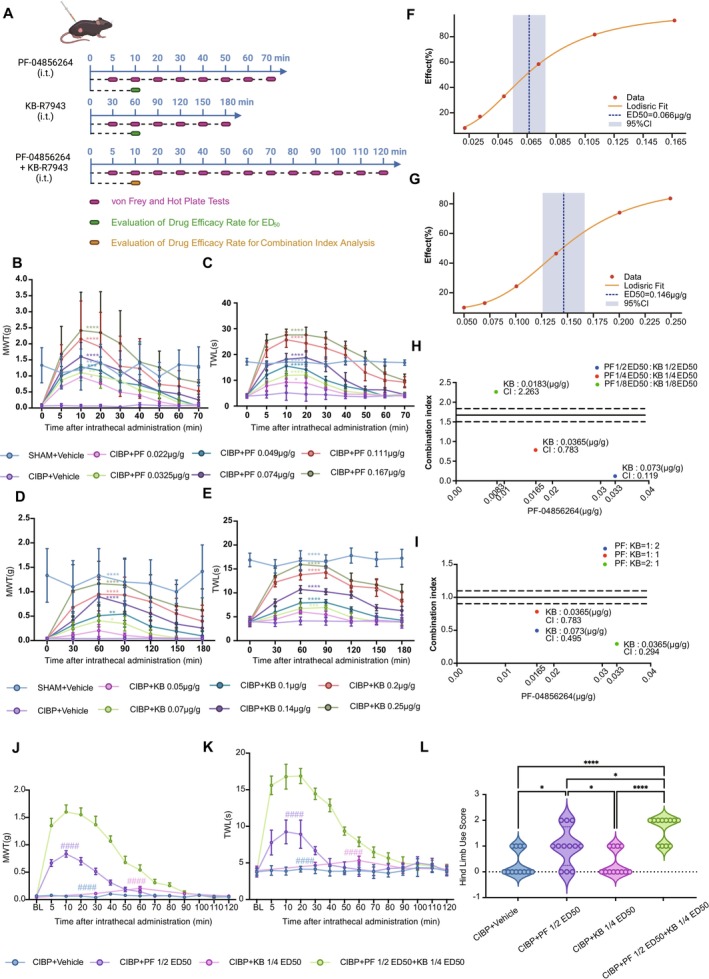
Analgesic effects and synergy of PF‐04856264 and KB‐R7943 in CIBP mice. (A) Experimental timeline for intrathecal administration of PF‐04856264, KB‐R7943, or their combination, followed by behavioral tests (von Frey and hot plate) at specified time points for ED_50_ and CI analysis. (B–D) Dose‐dependent analgesic effects of PF‐04856264 (PF) (0.022–0.167 μg/g) and KB‐R7943 (KB) (0.05–0.25 μg/g) on MWT and TWL. Statistical significance (* symbols) indicates differences in the AUC compared with the CIBP+ Vehicle group. (F, G) Dose–response curves and ED_50_ determination for PF (F) and KB (G) were obtained through logistic regression fitting. (H) CI analysis revealed significant synergistic effects at medium and high doses (1/4 and 1/2 ED_50_), with antagonistic effects at low doses (1/8 ED_50_). (I) CI analysis at a fixed 1/4 ED_50_ dose baseline showed the strongest synergy at a 2:1 ratio (CI = 0.294). (J–L) Combination therapy significantly improved MWT, TWL, and hind limb use scores compared to single‐agent treatments. Statistical significance (# symbols) indicates differences in the AUC compared with the CIBP + PF 1/2 ED_50_ + KB 1/4 ED_50_ group. All data were tested for normality using the Shapiro–Wilk test and are presented as mean ± SEM. Statistical tests: (B–E, J–K) One‐way ANOVA with Bonferroni post hoc test; (L) Kruskal–Wallis test with Dunn's post hoc test; (F, G) Four‐parameter logistic regression with 95% confidence intervals derived from the covariance matrix; (H, I) Combination Index calculation using the Chou‐Talalay method with dose‐effect curve interpolation via the SciPy library (fsolve function). (*p < 0.05, **p < 0.01, ***p < 0.001, ****p < 0.0001 versus the CIBP + vehicle group; ^#^ *p* < 0.05, ^##^ *p* < 0.01, ^###^ *p* < 0.001, ^####^ *p* < 0.0001 versus the CIBP + PF 1/2 ED_50_ + KB 1/4 ED_50_ group; *p < 0.05, **p < 0.01, ***p < 0.001, ****p < 0.0001 for comparisons between experimental groups). *n* = 12 per group for (B–E, H–L); *n* = 11–15 per group for (F, G). i.t., intrathecal injection; ED_50_, half‐maximal effective dose; AUC, area under the curve; CI, combination index.

L4‐6 DRG tissues were harvested on day 21 for further analysis (Figure 1H). mRNA expression levels of VGSCs and NCX family members in the L4‐6 DRGs of sham‐operated mice were first assessed at baseline. qPCR results revealed that Nav1.7 and Nav1.8 were the predominant VGSC subtypes in the DRG, with Nav1.8 exhibiting slightly higher expression than Nav1.7. Among the NCX family members, NCX1 had the highest expression, significantly exceeding that of NCX2 and NCX3 (Figure 1I,J). In CIBP mice, mRNA levels of VGSC and NCX subtypes were generally elevated, with Nav1.7 and NCX1 exhibiting the most pronounced increases (Figure 2K,L). Western blot analysis further confirmed the significant increase in Nav1.7 and NCX1 protein levels (Figure 1M,N), suggesting that these two subtypes may play key roles in CIBP‐associated pain mechanisms. Dual immunofluorescence staining and fluorescence intensity analysis demonstrated a high degree of colocalization between Nav1.7 and NCX1 in DRG neurons (Figure 1O,P), with a Pearson correlation coefficient of 0.809 ± 0.048. This spatial distribution pattern may provide a structural foundation for the functional interaction between Nav1.7 and NCX1 in pain signal transmission.

### 3.3 Analgesic Effects and Synergism of Intrathecal Nav1.7 and NCX1 Inhibitors in CIBP Mice

To further explore the impact of targeting Nav1.7 and NCX1 on pain sensitivity in CIBP mice, we systematically assessed the analgesic effects of PF‐04856264 (a Nav1.7‐specific inhibitor) and KB‐R7943 (an NCX1 reverse‐mode inhibitor) at multiple time points via behavioral tests. In addition, we calculated the ED_50_ and combination index (CI). These drugs were administered via intrathecal injection and evaluated both as monotherapy and in combination (Figure 2A).

Both PF‐04856264 (0.022–0.167 μg/g) and KB‐R7943 (0.05–0.25 μg/g) dose‐dependently increased the MWT and TWL, with PF‐04856264 exhibiting greater analgesic potency but a shorter duration of action (Figure 2B–E). The calculated ED50 values for PF‐04856264 and KB‐R7943 were 0.066 ± 0.006 μg/g (95% CI: 0.055–0.078 μg/g) and 0.146 ± 0.010 μg/g (95% CI: 0.126–0.167 μg/g), respectively (Figure 2F,G).

For combination treatments, the coadministration of PF‐04856264 and KB‐R7943 at 1/2ED_50_ and 1/4ED_50_ baseline doses (1:1 ratio) significantly amplified the analgesic effects, yielding combination index (CI) values of 0.119 and 0.783, respectively, indicating strong synergy. However, at 1/8ED_50_, the CI value increased to 2.263, suggesting antagonism (Figure 2H). These findings highlight that the analgesic effects of PF‐04856264 and KB‐R7943 in combination are dose dependent, emphasizing the critical importance of optimizing drug ratios for maximal therapeutic efficacy.

To assess the influence of drug ratios on analgesia, we tested PF‐04856264 and KB‐R7943 at 1/2ED_50_ baseline doses at ratios of 1:1, 1:2, and 2:1. While the 1:2 and 2:1 ratios resulted in motor impairment, likely due to neurotoxicity or off‐target effects at higher doses, testing at the 1/4ED_50_ baseline dose revealed that the 2:1 ratio demonstrated the strongest synergy (CI: 0.294) without inducing motor impairment (Figure 2I). Moreover, at the 1/4ED_50_ dose, the 2:1 combination not only significantly prolonged the analgesic duration and enhanced the analgesic effects, resulting in greater efficacy than single‐agent treatments but also effectively alleviated movement‐related pain in CIBP, as evidenced by a significant increase in hind limb use scores (Figure 2J–L).

### 3.4 Combined Inhibition of Nav1.7 and NCX1 Reduces Calcium Overload in Primary Sensory Neurons Stimulated by Veratridine

**FIGURE 3:**
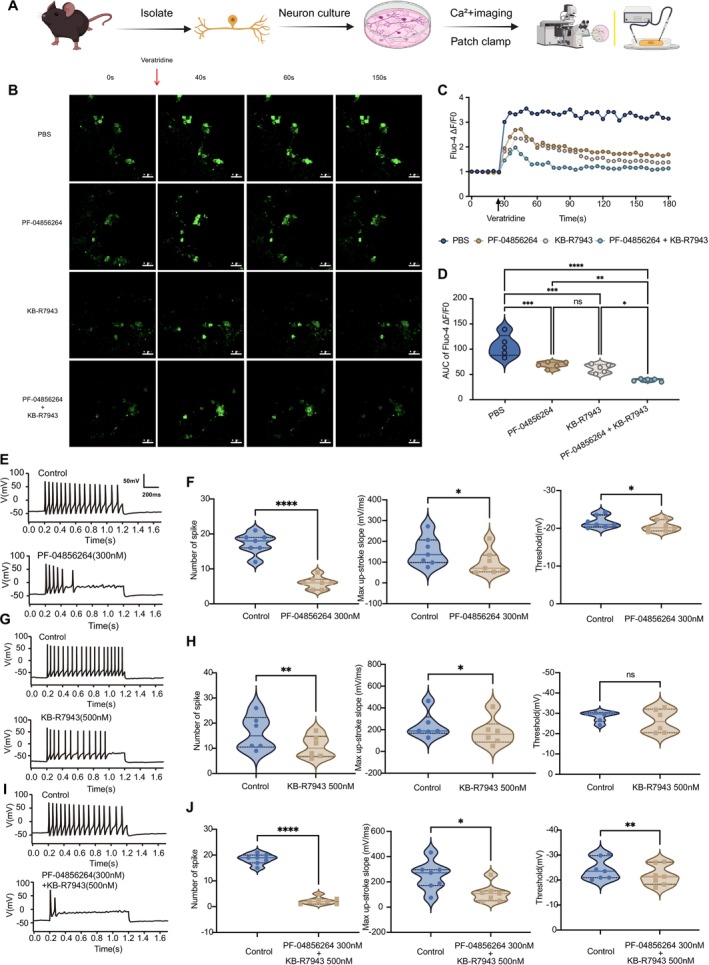
Effects of PF‐04856264, KB‐R7943, and their combination on calcium signaling and excitability in DRG neurons. (A) Schematic of the experimental workflow showing DRG neuron isolation, in vitro culture, calcium imaging, and patch‐clamp recordings to evaluate the effects of PF‐04856264, KB‐R7943, and their combination. (B–D) Calcium imaging: (B) Representative confocal microscopy images of calcium fluorescence intensity (Fluo‐4 AM, Δ*F*/*F*_0_) showing the response of DRG neurons to veratridine stimulation (30 μM) in each treatment group. (C) Time course of Fluo‐4 fluorescence intensity (Δ*F*/*F*_0_) over 180 s, showing dynamic changes in intracellular calcium levels following veratridine stimulation. Drug treatments attenuated calcium influx, with the combination exhibiting the strongest reduction. Data are normalized to baseline (Δ*F*/*F*_0_ at *t* = 0). (D) Area under the curve (AUC) analysis of calcium signals from (C), quantifying cumulative intracellular calcium influx over the recording period. Drug treatment conditions for calcium imaging: DRG neurons were preincubated with PF‐04856264 (2 μM), KB‐R7943 (5 μM), or their combination for 30 min prior to veratridine stimulation. (E–J) Patch‐clamp recordings: (E, G, I) Representative whole‐cell current‐clamp traces showing neuronal action potential firing in response to depolarizing current injection. Treatment with PF‐04856264 and KB‐R7943 reduced neuronal excitability, with the combination showing the strongest inhibition. The scale bar in (E) applies to all traces. (F, H, J) Quantitative analysis of action potential parameters, including the number of spikes, maximal upstroke slope (mV/ms), and action potential threshold (mV), across treatment groups. Drug treatment conditions for patch‐clamp recordings: PF‐04856264 (300 nM), KB‐R7943 (500 nM). All data were tested for normality using the Shapiro–Wilk test and are presented as mean ± SEM. Statistical tests: (D) One‐way ANOVA with Bonferroni post hoc test; (F, H, J) Paired Student's *t*‐test. (*p < 0.05, **p < 0.01, ***p < 0.001, ****p < 0.0001). *n* = 5 per group for (B–D); *n* = 6–7 per group for (E–J).

We conducted calcium imaging experiments by utilizing the voltage‐gated sodium channel (VGSC) agonist veratridine to stimulate primary sensory neurons isolated and cultured from the DRG of mice, aiming to simulate the effects of VGSC activation on sodium‐calcium balance in the CIBP model (Figure 3A). By employing PF‐04856264 and KB‐R7943 to inhibit Nav1.7‐mediated sodium influx and NCX1 reverse transport (calcium influx and sodium efflux), respectively, we observed changes in calcium fluorescence intensity in small‐to‐medium diameter (diameter < 30 μm) nociceptive sensory neurons. This finding allowed us to further investigate the regulatory roles of Nav1.7 and NCX1 in calcium homeostasis within DRG neurons.

Our results (Figure 3B–D) revealed that following veratridine‐induced VGSC activation, the calcium fluorescence intensity in the DRG neurons of the PBS group significantly increased, indicating substantial intracellular calcium accumulation. Inhibition of NCX1 reverse transport alone significantly reduced neuronal calcium fluorescence intensity (***p < 0.001), suggesting that NCX1 reverse‐mode activity may be a key mechanism contributing to intracellular calcium overload following VGSC activation. Similarly, the selective inhibition of Nav1.7 also significantly reduced the calcium fluorescence intensity (***p < 0.001), further indicating that Nav1.7‐mediated sodium influx may be an important driving force influencing NCX1 reverse‐mode transport. Notably, co‐administration of PF‐04856264 and KB‐R7943 resulted in the most pronounced reduction in calcium fluorescence intensity (****p < 0.0001), surpassing the effect of either inhibitor alone. Combined with the findings demonstrating the synergistic analgesic effects of dual inhibition, these calcium imaging results provide mechanistic support at the cellular level, suggesting that Nav1.7 and NCX1 may cooperatively regulate sodium–calcium homeostasis in DRG neurons, thereby contributing to pain sensitization in CIBP.

### 3.5 Combined Inhibition of Nav1.7 and NCX1 Reduces Hyperexcitability of Primary Sensory Neurons

We employed patch‐clamp electrophysiology to evaluate the effects of pharmacological inhibition of Nav1.7 and NCX1 on the excitability of primary mouse neurons (Figure 3A). Representative action potential trajectories (Figure 3E,G,I) and quantitative analyses of key parameters (Figure 3F,H,J) revealed that under 1.5‐ to 2‐fold threshold stimulation, both PF‐04856264 (300 nM) and KB‐R7943 (500 nM) alone effectively reduced the frequency of action potentials (PF: Cohen's *d* = 4.93, 95% CI: −13.40 to −9.169; KB: Cohen's *d* = 2.19, 95% CI: −8.134 to −2.866), with the combined treatment producing a more pronounced reduction (Cohen's *d* = 7.63, 95% CI: −18.10 to −14.19). Similarly, maximum up‐stroke slope was significantly decreased by PF‐04856264 (Cohen's *d* = 1.55, 95% CI: −87.04 to −22.12) and KB‐R7943 (Cohen's *d* = 1.09, 95% CI: −111.2 to −1.967), with the combined treatment leading to an even greater reduction (Cohen's *d* = 1.83, 95% CI: −205.4 to −67.63). Additionally, PF‐04856264 slightly increased the action potential threshold (Cohen's *d* = 1.13, 95% CI: 0.1935 to 1.935), whereas KB‐R7943 alone did not produce a significant effect (Cohen's *d* = 0.57, 95% CI: −1.895 to 6.401). However, the combination of PF‐04856264 and KB‐R7943 resulted in a substantial increase in threshold potential (Cohen's *d* = 2.04, 95% CI: 1.326 to 3.519), stabilizing the neuronal membrane and reducing its susceptibility to activation. These findings suggest that dual inhibition of Nav1.7 and NCX1 reduces sensory neuron excitability, enhances membrane stability, and consequently attenuates the transmission and amplification of pain signals. The functional interaction between Nav1.7 and NCX1 in regulating neuronal excitability may contribute to their synergistic analgesic effects on CIBP.

### 3.6 Combined Inhibition of Nav1.7 and NCX1 Reduces the Neuroinflammatory Response in the DRGs of CIBP Mice

**FIGURE 4:**
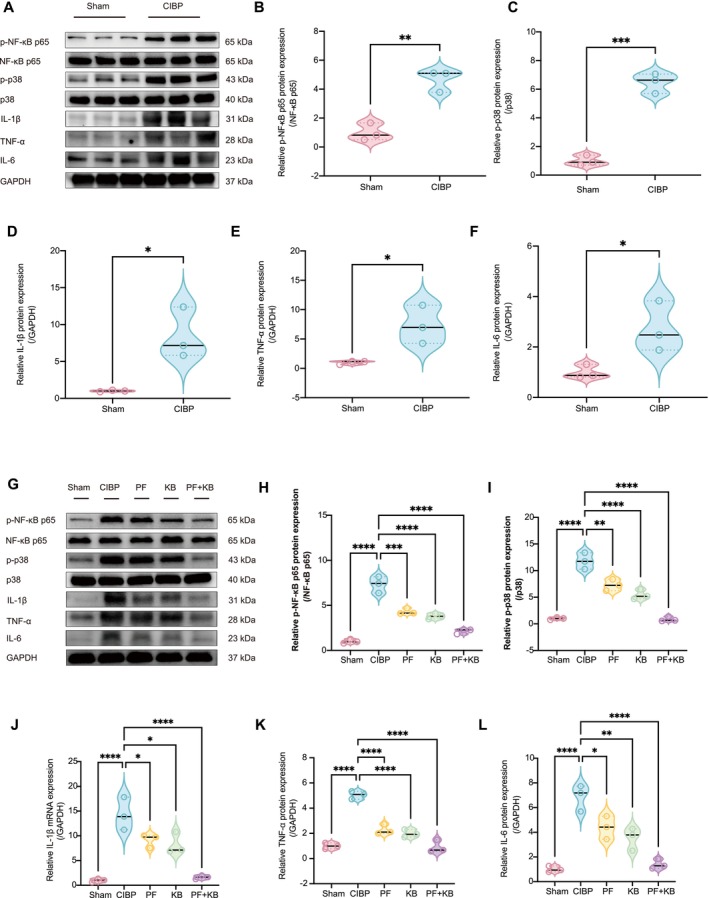
Dual inhibition of Nav1.7 and NCX1 attenuates neuroinflammatory responses in the L4–L6 DRGs of CIBP mice. (A) Representative Western blot images showing increased phosphorylation of NF‐κB p65 (p‐NF‐κB p65) and p38 MAPK (p‐p38), along with elevated levels of proinflammatory cytokines (IL‐1β, TNF‐α, IL‐6) in the L4–L6 DRGs of CIBP mice compared to sham controls. (B–F) Quantitative analysis of the Western blot data demonstrating significant upregulation of p‐NF‐κB p65 (B), p‐p38 (C), IL‐1β (D), TNF‐α (E), and IL‐6 (F) in CIBP mice relative to sham controls. (G) Representative immunoblots of DRG tissue from sham, CIBP, PF‐04856264 (PF, Nav1.7 inhibitor), KB‐R7943 (KB, NCX1 reverse‐mode inhibitor), and combined (PF + KB) treatment groups. (H–L) Quantification shows that both PF and KB monotherapies significantly reduced p‐NF‐κB p65 (H), p‐p38 (I), IL‐1β (J), TNF‐α (K), and IL‐6 (L) expression, whereas the combination therapy achieved the most significant reduction in these inflammatory markers. All data were tested for normality using the Shapiro–Wilk test and are presented as mean ± SEM. Statistical tests: (B–F) Unpaired Student's *t*‐test; (H–L) One‐way ANOVA with Bonferroni post hoc test. (*p < 0.05, **p < 0.01, ***p < 0.001, ****p < 0.0001). *n* = 3 per group.

Our findings showed that, compared with the sham‐operated control group, the phosphorylation levels of p38 MAPK and NF‐κB p65 proteins were significantly elevated in the L4–L6 DRGs of CIBP mice (Figure 4A–C), accompanied by increased protein expression of proinflammatory cytokines, including IL‐1β, TNF‐α, and IL‐6 (Figure 4A,D–F). These results indicate that the activation of the p38 MAPK/NF‐κB signaling pathway is involved in the neuroinflammatory response associated with CIBP.

Intrathecal administration of PF‐04856264 (a Nav1.7 inhibitor) or KB‐R7943 (a reverse‐mode NCX1 inhibitor), either alone or in combination, significantly reduced phosphorylation of p38 MAPK and NF‐κB p65 (Figure 4G–I), as well as the protein expression of proinflammatory cytokines in the DRG (Figure 4G,J–L). Notably, combined treatment resulted in a more pronounced suppression of these inflammatory markers compared to single‐agent administration.

These findings indicate that targeting Nav1.7 and NCX1 effectively attenuates neuroinflammatory responses in the DRGs of CIBP mice, with dual inhibition exerting a greater suppressive effect on p38 MAPK/NF‐κB signaling and proinflammatory cytokine expression.

### 3.7 Knockdown of Nav1.7 and NCX1 Mitigates Neuronal Inflammatory Responses in ND7‐23 Cells

The ND7‐23 cell line, a hybrid of mouse neuroblastoma cells and rat dorsal root ganglion (DRG) neurons, expresses key neuronal ion channels and exhibits physiological properties similar to those of primary DRG neurons [36]. To minimize confounding effects from non‐neuronal cell types and reduce potential off‐target drug effects, we employed shRNA‐mediated knockdown of Nav1.7 and NCX1 individually or in combination to examine their roles in regulating neuronal inflammatory responses.

**FIGURE 5:**
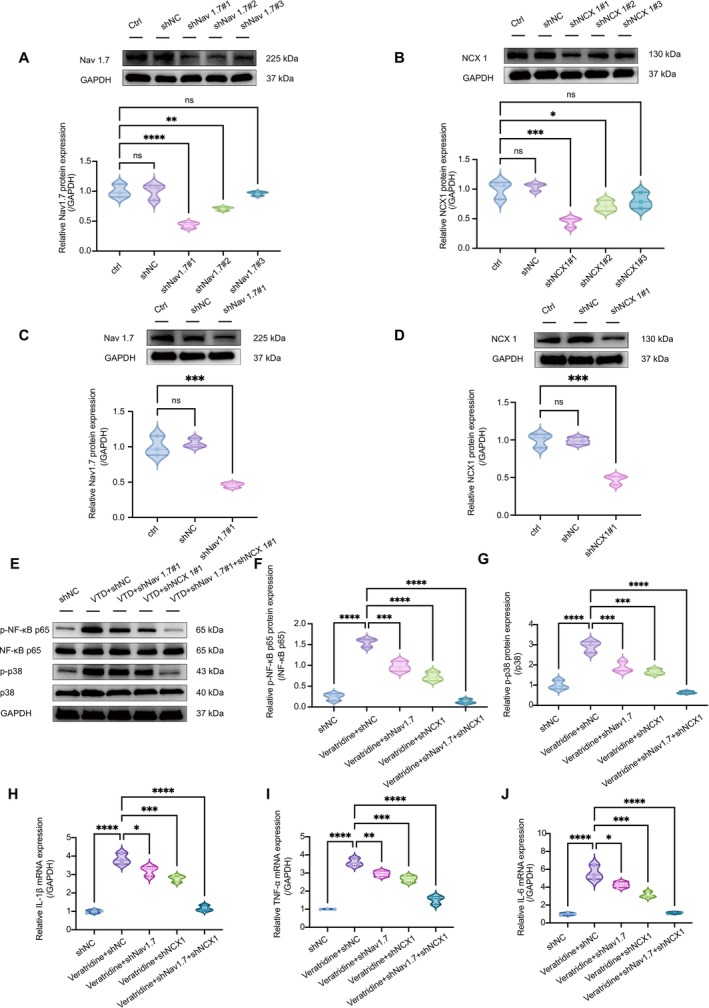
Dual knockdown of Nav1.7 and NCX1 synergistically suppresses veratridine‐induced p38 MAPK/NF‐κB activation and proinflammatory cytokine expression in ND7‐23 Cells. (A, B) Western blot analysis confirmed effective knockdown of Nav1.7 and NCX1 using shNav1.7#1 and shNCX1#1, respectively. Protein levels were reduced by approximately 57% for Nav1.7 and 55% for NCX1 compared to controls. (C, D) Combined knockdown using shNav1.7#1 and shNCX1#1 significantly reduced protein expression of both targets in ND7‐23 cells, with knockdown efficiencies of approximately 53% for Nav1.7 and 52% for NCX1. (E–G) Upon 24 h stimulation with veratridine (10 μM), both single and combined knockdown markedly suppressed phosphorylation of NF‐κB p65 and p38 MAPK. Notably, dual knockdown produced a stronger inhibitory effect, suggesting a synergistic interaction. (H–J) Similarly, veratridine‐induced mRNA expression of IL‐1β (H), TNF‐α (I), and IL‐6 (J) was significantly reduced in knockdown groups, with the most pronounced reduction observed in the dual knockdown condition. All data were tested for normality using the Shapiro–Wilk test and are presented as mean ± SEM. Statistical tests: (A–D, F–J) One‐way ANOVA with Bonferroni post hoc test. (*p < 0.05, **p < 0.01, ***p < 0.001, ****p < 0.0001). *n* = 3 per group.

Western blot analysis confirmed efficient knockdown using shNav1.7#1 and shNCX1#1. Nav1.7 and NCX1 protein levels were reduced by 57% and 55%, respectively, in the single‐knockdown groups, and by 53% and 52%, respectively, in the combined‐knockdown group, as quantified by densitometry analysis (Figure 5A–D).

To mimic excessive Nav1.7 activation observed in CIBP models and to induce an inflammatory response, ND7‐23 cells were stimulated with 10 μM veratridine for 24 h. Following stimulation, both single and combined knockdown significantly attenuated p38 MAPK and NF‐κB phosphorylation (Figure 5E–G), as well as the mRNA expression of IL‐1β, TNF‐α, and IL‐6 (Figure 5H–J). Notably, the combined knockdown exhibited a more pronounced inhibitory effect compared to individual knockdowns, indicating an enhanced combinatorial effect of Nav1.7 and NCX1 on neuroinflammatory responses.

## 4 Discussion

**FIGURE 6:**
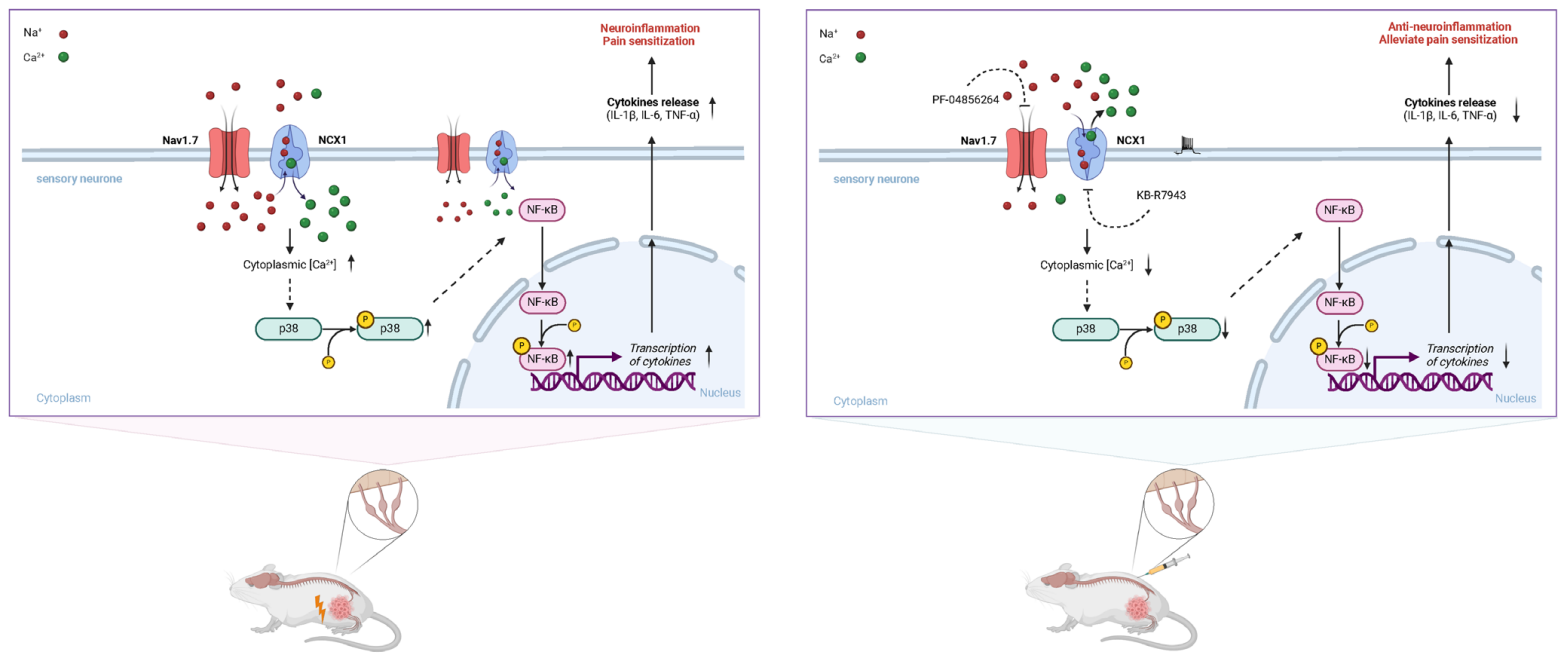
Mechanistic illustration of Nav1.7 and NCX1 in CIBP‐associated neuroinflammation and pain sensitization, and the therapeutic effects of dual‐target inhibition. Left panel: Pathological mechanisms of CIBP. In the DRG sensory neurons of CIBP mice, Nav1.7 mediates sodium (Na^+^) influx, leading to sodium overload and driving NCX1 into reverse mode. This reverse operation causes the accumulation of intracellular calcium (Ca^2+^). Elevated cytoplasmic calcium levels activate the p38 MAPK signaling pathway and induce the nuclear translocation and phosphorylation of NF‐κB, promoting the transcription of proinflammatory cytokines (IL‐1β, IL‐6, and TNF‐α). These cytokines exacerbate neuroinflammation and pain sensitization. Right panel: Therapeutic effects of dual‐target inhibition. The Nav1.7 inhibitor PF‐04856264 and the NCX1 reverse‐mode inhibitor KB‐R7943 reduce sodium influx and calcium overload, respectively, decreasing the excitability of sensory neurons. By blocking the activation of the p38 MAPK/NF‐κB signaling pathway, the transcription and release of proinflammatory cytokines are significantly suppressed. Through the aforementioned mechanism, the dual‐target strategy effectively alleviates neuroinflammation and pain sensitization. The dashed line between Ca^2+^ and p38 MAPK represents a hypothetical regulatory link, as our experimental data have not directly confirmed the regulation of p38 MAPK by Ca^2+^.

This study reveals the pivotal role of Nav1.7 and NCX1 in pain sensitization and neuroinflammation in CIBP and provides experimental evidence supporting their functional interaction. Our study provides the first evidence that Nav1.7 and NCX1 are significantly upregulated and highly colocalized in the L4–6 dorsal root ganglia (DRGs) of CIBP mice, suggesting that these two ion channels may contribute to the progression of CIBP and exhibit a potential functional interplay. Behavioral experiments indicate that the dual inhibition of Nav1.7 and NCX1 produces a significant synergistic analgesic effect in CIBP model mice. Mechanistic investigations indicate that Nav1.7 and NCX1 play a role in regulating sensory neuronal excitability and calcium homeostasis, which in turn modulates pain sensitization and neuroinflammatory responses. Compared with single‐target inhibition, dual‐target inhibition not only enhances analgesic efficacy but also more strongly suppresses neuroinflammation, particularly through modulation of the p38 MAPK/NF‐κB signaling pathway (Figure 6).

The novelty of this study lies in the first identification of the functional coupling between Nav1.7 and NCX1 in the DRG neurons of CIBP mice and the demonstration of the significant advantages of dual‐target inhibition in alleviating pain and suppressing neuroinflammatory responses. These findings offer new potential targets and intervention strategies for treating CIBP, while providing a theoretical foundation for the development of effective multitarget combination therapies.

Nav1.7 and NCX1 play critical roles in regulating neuronal excitability and calcium homeostasis, making them key modulators of the pathophysiology of CIBP. This study identified Nav1.7 as a threshold channel that, owing to its slow closed‐state inactivation properties, is highly sensitive to small‐amplitude and slow subthreshold stimuli. During subthreshold depolarization, Nav1.7 facilitates sodium influx, significantly enhancing neuronal excitability [37, 38, 39]. This mechanism explains the mechanical and thermal hypersensitivity observed in CIBP mice with upregulated Nav1.7 expression. In contrast, the inhibition of Nav1.7 reduces sodium influx during the early phase of depolarization, as evidenced by decreased neuronal excitability in patch‐clamp recordings and the resulting analgesic effects observed in behavioral experiments.

NCX1 primarily regulates intracellular calcium concentrations through sodium‐calcium exchange. However, in CIBP models, the upregulation of VGSCs, including Nav1.7, results in sodium overload, driving NCX1 to operate in reverse mode. This reverse operation exacerbates intracellular calcium accumulation, which increases neuronal excitability and potentially activates calcium‐dependent signaling pathways, such as those involving protein kinase C (PKC) and calcium/calmodulin‐dependent protein kinase II (CaMKII). These pathways further increase VGSC activity through positive feedback mechanisms, perpetuating pain sensitization [40]. Consequently, inhibiting the reverse transport of NCX1 can effectively alleviate calcium overload, reduce neuronal excitability, and potentially interrupt the positive feedback loop that amplifies pain signaling.

In recent years, proximity ligation assay (PLA) techniques have revealed that Nav1.6 and NCX are located in extremely close spatial proximity (< 40 nm) within the T‐tubules of cardiomyocytes, forming unique nanodomains that facilitate efficient intracellular ion transport [41, 42]. In this study, we observed a Pearson colocalization coefficient of 0.809 ± 0.048 between Nav1.7 and NCX1 in the DRG neurons of CIBP mice, indicating a significant spatial association between these two proteins, which is consistent with previous findings. This close spatial proximity provides a structural foundation for the functional coupling between Nav1.7‐mediated sodium influx and NCX1 sodium–calcium exchange activity. Such coupling likely coordinates ion flux, thereby regulating sodium–calcium homeostasis and neuronal excitability.

Interestingly, similar VGSC–NCX functional coupling mechanisms have been validated in various disease models. For instance, in Alzheimer's disease, the interaction between Nav1.6 and NCX3 helps maintain intracellular calcium homeostasis, protecting neurons from calcium overload‐induced damage [31]. In small‐fiber neuropathy, axonal degeneration caused by gain‐of‐function mutations in Nav1.7 can be reversed by inhibiting the reverse mode of NCX [43, 44]. More recently, a novel Nav1.7–NCX interaction has been identified beyond excitable neurons, particularly in chondrocytes, where Nav1.7 regulates intracellular calcium signaling through NCX, contributing to osteoarthritis progression and pain hypersensitivity [45]. These findings suggest that VGSC–NCX functional coupling is a conserved mechanism across multiple disease models, providing a theoretical basis for the observed Nav1.7–NCX1 coupling in CIBP mice. Our study extends this mechanism to the context of pain sensitization and neuroinflammation, further highlighting its potential significance in chronic pain.

However, multiple isoforms of the VGSC and NCX families are expressed in DRG, and whether this functional coupling is specific to Nav1.7 and NCX1 remains to be further explored. Within the VGSC family, Nav1.8 and Nav1.9 are also expressed in peripheral sensory neurons and contribute to pain signal transmission. However, Nav1.8 primarily facilitates action potential conduction rather than the initiation of pain sensitization, while Nav1.9, with its relatively low expression, mainly regulates the resting membrane potential. Their distinct electrophysiological properties make it unlikely for them to exhibit the same threshold sensitivity and amplification effects as Nav1.7 in the context of CIBP.

Similarly, in the NCX family, the expression levels of different isoforms in DRG also vary significantly. qPCR results indicate that NCX1 is the predominant isoform in DRG neurons of CIBP mice, with significantly higher expression than NCX2 and NCX3. Although NCX3 has been shown to exhibit neuroprotective effects in certain neurodegenerative diseases, such as Alzheimer's disease, it is primarily expressed in central neurons, whereas its expression in DRG is much lower than that of NCX1. Therefore, this indicates that Nav1.7 and NCX1 play a specific regulatory role in CIBP and make a unique contribution to its associated pain hypersensitivity and neuroinflammation.

In animal behavioral experiments, we observed that at medium to high doses (1/2–1/4 ED_50_), the Nav1.7 inhibitor PF‐04856264 and the NCX1 reverse mode inhibitor KB‐R7943 exhibited significant synergistic effects, with combination indices (CIs) of 0.119 and 0.783, respectively. However, at low doses (1/8 ED_50_), the two drugs displayed antagonistic interactions, with a CI of 2.263. Furthermore, at a baseline dose of 1/4 ED_50_, different ratios of PF‐04856264 to KB‐R7943 (1:2, 1:1, and 2:1) demonstrated varying degrees of synergy, with CIs of 0.495, 0.783, and 0.294, respectively. This dose‐dependent synergistic or antagonistic effect is similar to the interactions observed in earlier studies on the inhibitory effects of morphine and clonidine on gastrointestinal motility [46]. These findings underscore the complexity of drug interactions and highlight the importance of optimizing dosage and drug ratios to achieve maximal therapeutic efficacy.

Our study has significant clinical implications, demonstrating that multitarget combination therapy can reduce the dosage of individual drugs, thereby minimizing side effects and improving the safety and tolerability of treatments. This mechanism of action has potential applications not only in the context of CIBP but also in other chronic pain conditions, such as diabetic neuropathy and inflammatory pain, indicating broader therapeutic potential.

Neuroinflammation is one of the key pathological mechanisms promoting the development of CIBP. It primarily facilitates disease progression by increasing pain sensitization, inducing neural remodeling, and maintaining persistent inflammatory feedback [4]. Previous studies have predominantly attributed neuroinflammation to the actions of immune cells, such as microglia and astrocytes [47]. However, recent research has indicated that neuronal inflammatory responses, as critical components of neuroinflammation, facilitate pain sensitization and the progression of chronic pain [48, 49].

Neurons play an active role in neuroinflammation, not only responding passively to inflammatory stimuli but also actively regulating the inflammatory response through the secretion of proinflammatory factors. This process may involve their intrinsic “immune‐like” functions. DRG neurons express pattern recognition receptors (PRRs) and proinflammatory signaling pathways (such as TLRs, NF‐κB, and MAPKs), which can be activated under pathological conditions, leading to the transcription and secretion of proinflammatory factors. For example, following peripheral nerve injury, NF‐κB signaling is activated in DRG neurons, promoting the synthesis of TNF‐α, IL‐6, and other proinflammatory factors [50, 51]. These proinflammatory factors not only enhance neuronal excitability and regulate neuronal survival through autocrine signaling but also activate adjacent glial cells and neurons through paracrine signaling, thereby amplifying and sustaining neuropathic pain. Additionally, DRG neurons secrete CCL2, which promotes the recruitment of macrophages to the DRG, where these immune cells further release proinflammatory factors, forming a positive feedback loop that exacerbates inflammation [52]. These findings suggest that neurons are not merely passive targets of inflammation but actively shape the inflammatory microenvironment, playing a crucial role in pain conditions.

The functions of voltage‐gated sodium channels (VGSCs) and sodium‐calcium exchangers (NCXs) in various disease models are closely associated with the development of neuroinflammation. Nav1.7 has been shown to play a crucial role in inflammatory pain, as nociceptor‐specific deletion of Nav1.7 in mice significantly reduced or abolished pain responses to inflammatory stimuli such as formalin, carrageenan, CFA, and NGF, underscoring its importance in pain amplification [23]. Similarly, prolonged inflammation has been reported to alter NCX activity in nociceptive neurons, which may contribute to the development and maintenance of inflammatory pain [53].

Considering the involvement of Nav1.7 and NCX1 in inflammatory pain, their abnormal upregulation may play a critical role in pain‐related neuroinflammation. In this study, we observed that the upregulation of Nav1.7 and NCX1 was closely associated with the activation of the p38 MAPK/NF‐κB signaling pathway in CIBP mice. Compared with single‐target interventions, dual inhibition of Nav1.7 and NCX1 significantly suppressed the activation of the p38 MAPK/NF‐κB signaling pathway and reduced the secretion of downstream proinflammatory cytokines. These findings suggest that a dual‐target strategy may be a more effective approach for treating CIBP‐related neuroinflammation. By simultaneously inhibiting Nav1.7 and NCX1, this strategy not only alleviates pain hypersensitivity but also more effectively suppresses neuroinflammatory signaling, providing a promising therapeutic intervention for refractory cancer‐induced bone pain.

Despite the promising analgesic and anti‐neuroinflammatory potential of targeting Nav1.7 and NCX1, several challenges remain for their clinical translation. One of the most critical concerns is selectivity. Nav1.7 inhibitors must minimize off‐target effects on Nav1.5 (cardiac) and Nav1.4 (skeletal muscle) to prevent cardiac conduction block and muscle weakness. Although existing small‐molecule inhibitors, such as PF‐04856264, have demonstrated good selectivity in preclinical studies [54], their clinical safety still requires further validation. Similarly, NCX1 inhibitors, such as KB‐R7943 and SEA0400, have been reported to exhibit potential cardiac toxicity [55], highlighting the need for the development of more selective reverse‐mode inhibitors.

Additionally, pharmacokinetics and safety remain key factors influencing clinical application. Ensuring that both inhibitors maintain effective concentrations in peripheral neurons while minimizing systemic toxicity is crucial for optimizing therapeutic efficacy. Combination therapy at lower doses may enhance analgesic effects while reducing adverse effects associated with higher doses, such as cardiac complications or systemic paralysis. Future studies should also evaluate long‐term safety, particularly regarding the potential compensatory upregulation of other ion channels or adverse cardiovascular effects.

Given the challenges in pain management for cancer‐induced bone pain (CIBP), intrathecal administration remains a commonly used and effective clinical option, particularly in advanced disease stages, where it can improve the quality of life for patients with refractory pain. If more selective and better‐tolerated Nav1.7/NCX1 small‐molecule inhibitors can be developed in the future, these drugs may become viable alternatives for oral or intravenous (IV) administration, allowing for earlier intervention in cancer pain treatment and reducing long‐term opioid dependence and tolerance issues.

Our study has several limitations that warrant further discussion. First, although the pharmacological inhibitors PF‐04856264 and KB‐R7943 effectively target Nav1.7 and NCX1, their in vivo specificity has not been fully validated. To address this shortcoming, we knocked down Nav1.7 and NCX1 in ND7‐23 cells via shRNA to further confirm their roles in regulating neuronal inflammatory responses. However, future studies should incorporate tissue‐specific conditional knockout models in DRG neurons to provide stronger in vivo evidence of their functions. Second, our findings suggest that Nav1.7 and NCX1 may indirectly modulate the activity of the p38 MAPK/NF‐κB pathway through intracellular calcium regulation. However, we did not utilize calcium chelators or calcium ionophores to directly validate the involvement of calcium ions and calcium‐related signaling pathways in CIBP. Future research should further investigate the regulation of calcium signaling to gain a more comprehensive understanding of the pathological mechanisms underlying CIBP pain. Finally, our study focused primarily on the role of neurons in CIBP, without examining the potential interactions between glial cells, such as microglia and astrocytes, and neurons. Given the critical role of glial cells in neuroinflammation, future research should explore glia–neuron interactions, thereby providing a more holistic understanding of the pain microenvironment in CIBP.

## 5 Conclusions

In this study, we elucidate the pivotal roles of Nav1.7 and NCX1 in cancer‐induced bone pain (CIBP). Our findings indicate that Nav1.7 and NCX1 functionally interact to regulate sodium‐calcium homeostasis, neuronal excitability, and neuroinflammatory responses associated with CIBP. Furthermore, the combination index provides quantitative evidence supporting the synergistic analgesic effects achieved through the dual inhibition of Nav1.7 and NCX1. Future research should focus on refining dual‐target therapeutic strategies, assessing their efficacy in other cancer‐related pain conditions and evaluating their feasibility and safety in clinical applications.

## Author Contributions

Yan Feng and Fang Yan contributed equally as first authors, while Weian Zeng and Yang Huang jointly served as corresponding authors. Yan Feng, Fang Yan, Weian Zeng, Yang Huang, and Dongtai Chen designed the study. Yan Feng and Fang Yan carried out experiments, processed the data, and prepared the manuscript. Peizong Wang, Yan Yan, and Xiangnan Chen provided technical support and assisted with animal and cellular experiments. Qiang Li and Wei Xing conducted molecular experiments. All authors revised and approved the final manuscript.

## Ethics Statement

All procedures adhered to the NIH Guide for the Care and Use of Laboratory Animals and were approved by the Animal Care and Use Committee of Sun Yat‐sen University Cancer Centre (Approval No. L025501202302009).

## Conflicts of Interest

The authors declare no conflicts of interest.

## Supporting information


Data S1

Table S1

Table S2

## Data Availability

The raw data of the experiment were uploaded onto the Research Data Deposit (RDD) (https://www.researchdata.org.cn) with an RDD number of RDDB2025652982.
