# Supplementary material for: Synergistic Inhibition of Nav1.7 and NCX1: A Novel Strategy for Treating Cancer‐Induced Bone Pain by Modulating Pain Sensitization and Neuronal Inflammation
Source: CNS Neurosci Ther. 2025 Apr 18;31(4):e70389. doi: 10.1111/cns.70389 (PMC12007017; doi:10.1111/cns.70389)


-Full unedited blot for Figure 1M

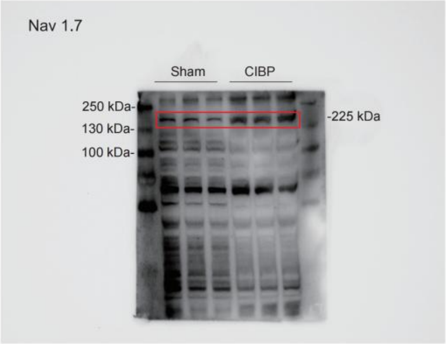

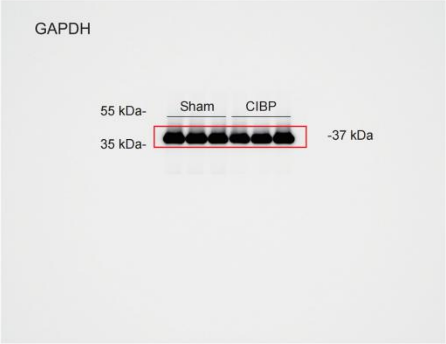

-Full unedited blot for Figure 1N

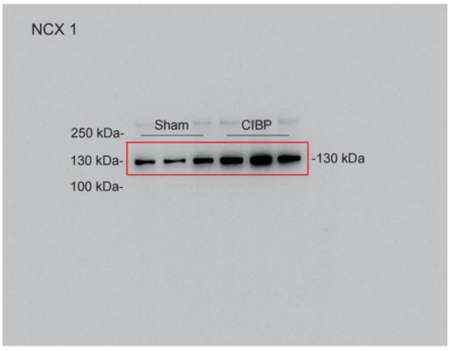

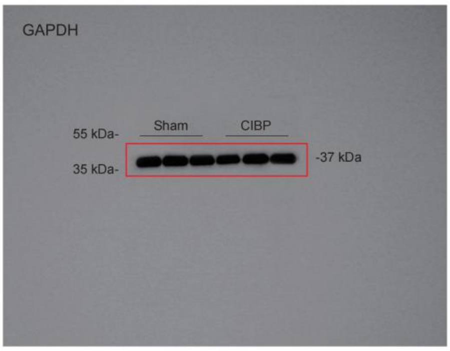

-Full unedited blot for Figure 4A

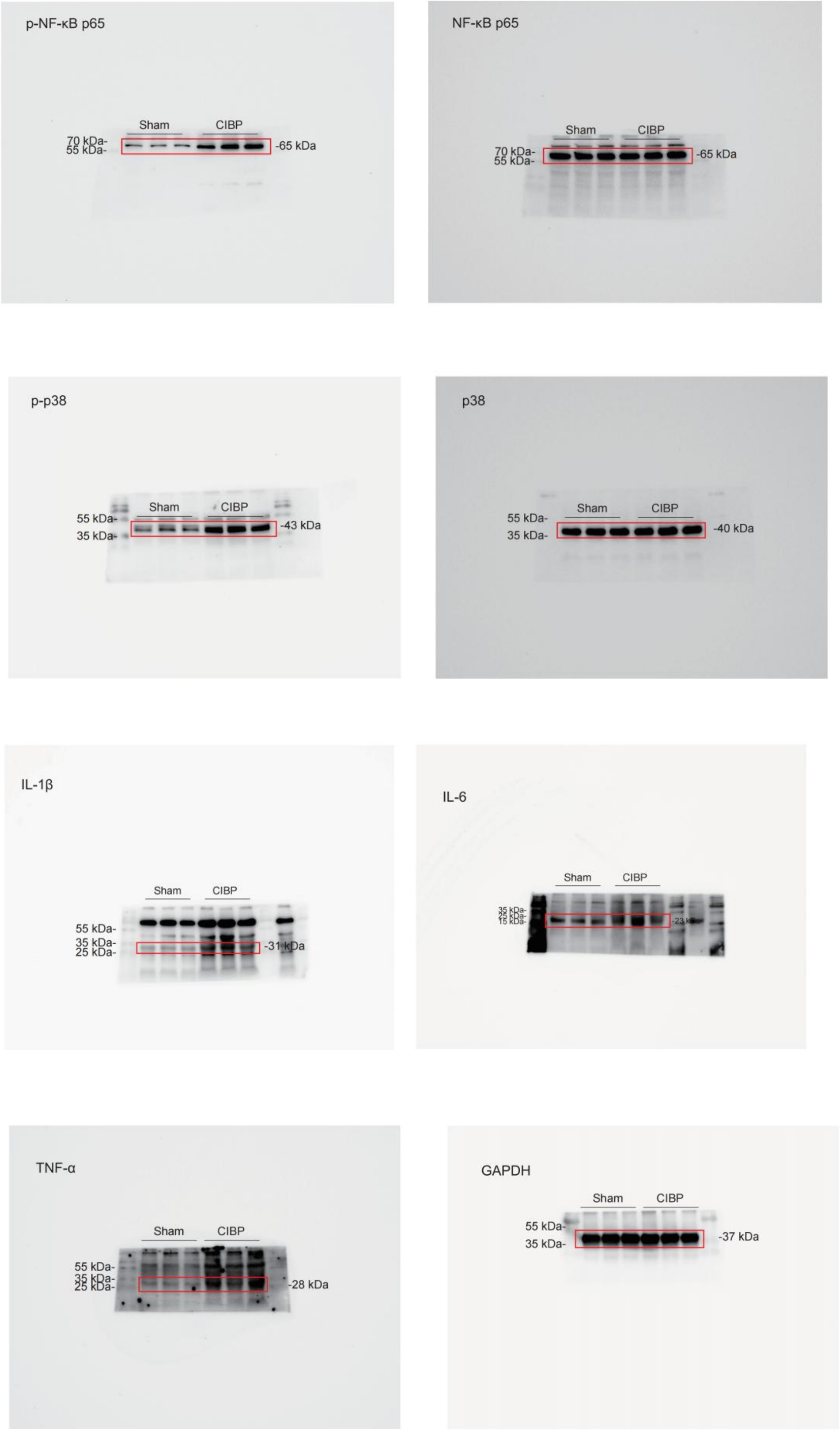

-Full unedited blot for Figure 4G

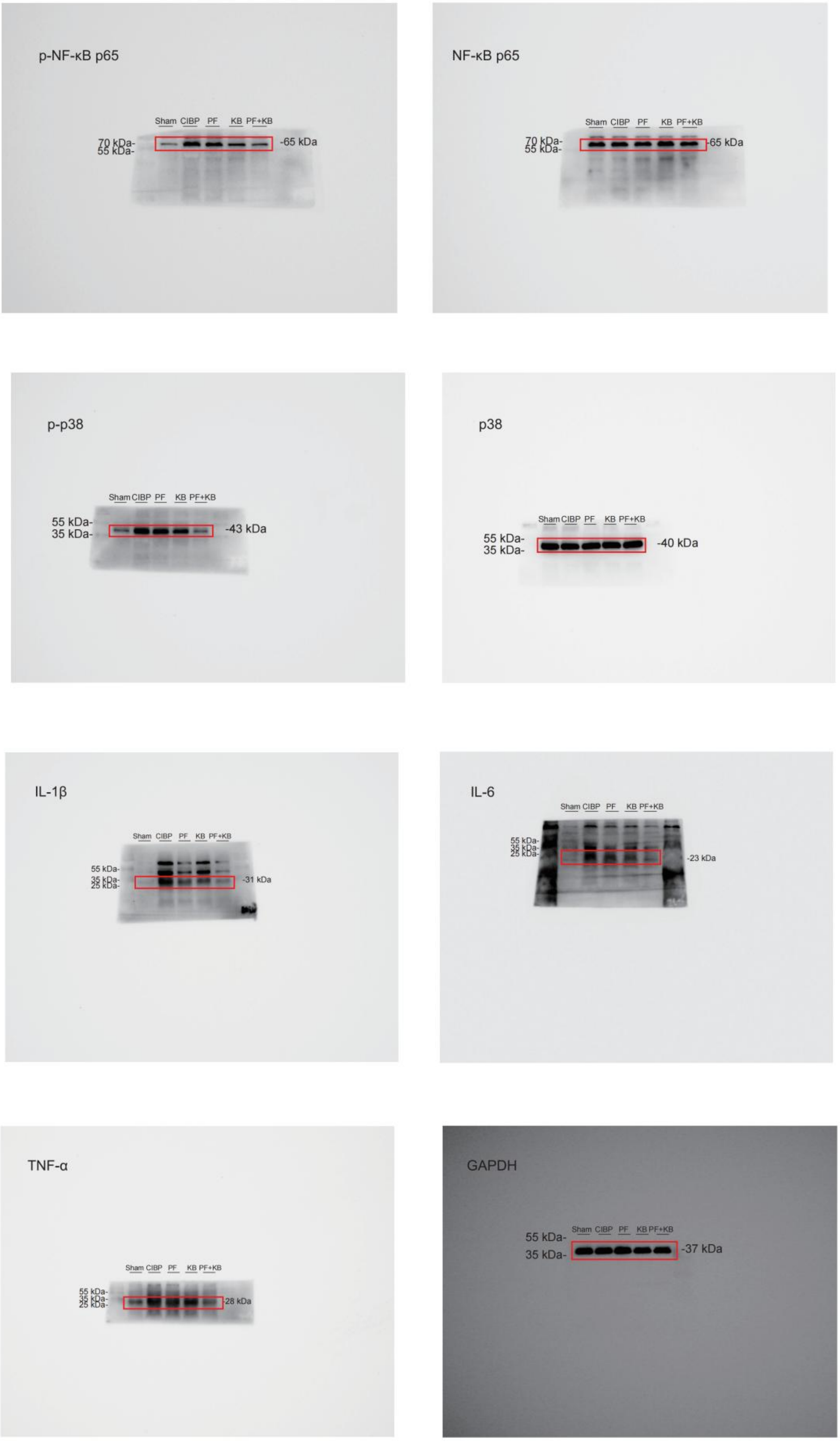

-Full unedited blot for Figure 5A

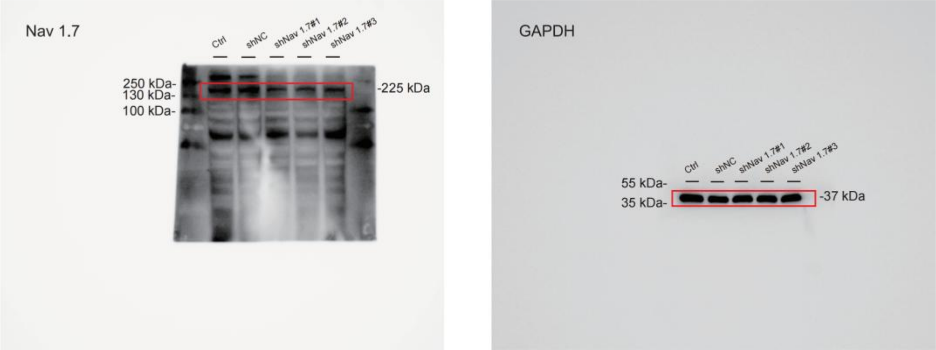

-Full unedited blot for Figure 5B

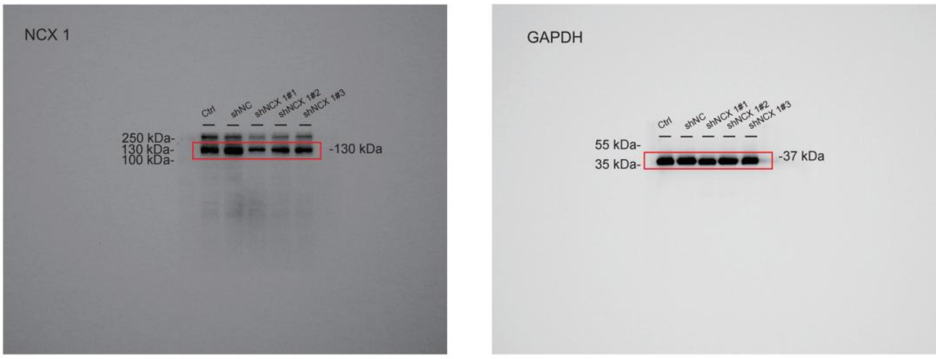

-Full unedited blot for Figure 5C

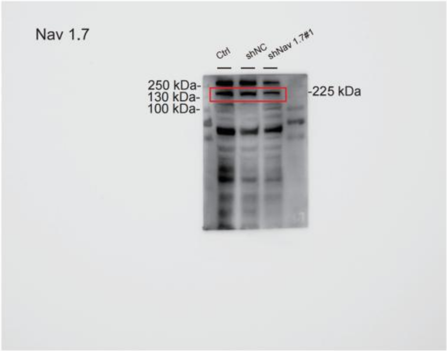

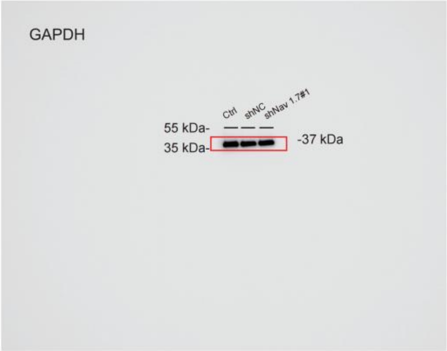

-Full unedited blot for Figure 5D

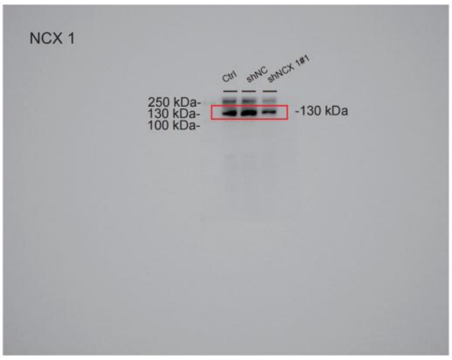

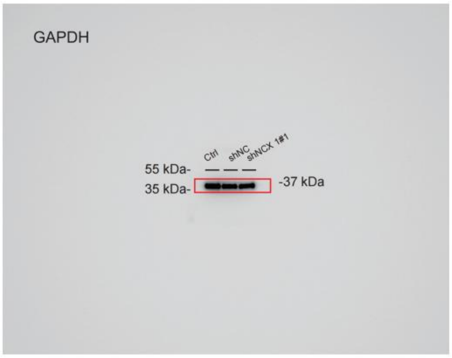

-Full unedited blot for Figure 5E

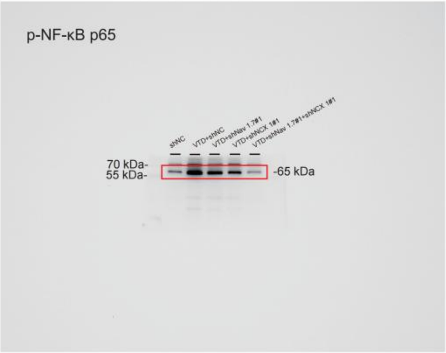

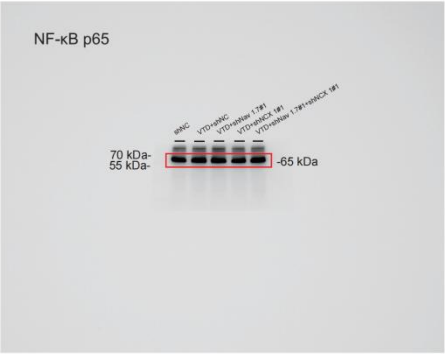

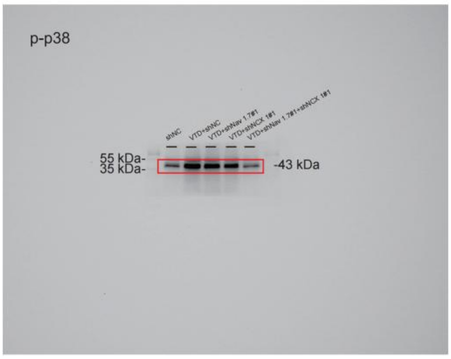

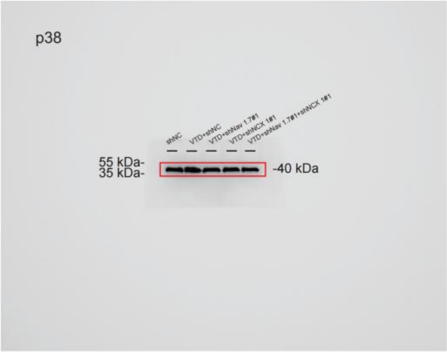

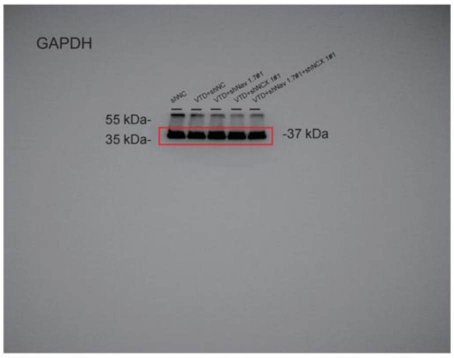

Supplement: Supplementary file 1 — Data S1 [file CNS-31-e70389-s002.pdf]
