# Supplementary material for: Synergistic Inhibition of Nav1.7 and NCX1: A Novel Strategy for Treating Cancer‐Induced Bone Pain by Modulating Pain Sensitization and Neuronal Inflammation
Source: CNS Neurosci Ther. 2025 Apr 18;31(4):e70389. doi: 10.1111/cns.70389 (PMC12007017; doi:10.1111/cns.70389)
Supplement: Supplementary file 2 — Table S1 [file CNS-31-e70389-s001.pdf]

Table S1. Information for primary antibodies

Name	Cat number	Source	Application
Rabbit anti-Nav1.7	ab65167	Abcam	WB: 1: 1000
Rabbit anti-NCX1	ab177952	Abcam	WB: 1: 6000
Rabbit anti-Phospho-NF-κB p65	3033	CST	WB: 1: 1000
Rabbit anti-NF-κB p65	8242	CST	WB: 1: 1000
Rabbit anti-Phospho-p38 MAPK	4511	CST	WB: 1: 1000
Rabbit anti-p38 MAPK	9212	CST	WB: 1: 1000
Rabbit anti- IL-1β	ab234437	Abcam	WB: 1: 1000
Rabbit anti-TNF-α	ab183218	Abcam	WB: 1: 1000
Rabbit anti-IL-6	ab259341	Abcam	WB: 1: 1000
Rabbit anti-GAPDH	380626	Zenbio	WB: 1: 5000
Rabbit anti-Nav1.7	ab65167	Abcam	IF: 1: 150
Mouse anti-NCX1	MA3926	Invitrogen	IF: 1: 100

Abbreviations: Nav1.7, Sodium Voltage-Gated Channel Alpha Subunit 9; NCX1, Sodium/Calcium Exchanger 1; Phospho-NF-κB p65, Phosphorylated Nuclear Factor Kappa B p65; NF-κB p65, Nuclear Factor Kappa B p65; Phospho-p38 MAPK, Phosphorylated p38 Mitogen-Activated Protein Kinase; p38 MAPK, p38 Mitogen-Activated Protein Kinase; IL-1β, Interleukin-1 Beta; TNF-α, Tumor Necrosis Factor Alpha; IL-6, Interleukin-6; GAPDH, Glyceraldehyde-3-Phosphate Dehydrogenase; IF, immunofluorescenc; WB, western blot.
