# Supplementary material for: Synergistic Inhibition of Nav1.7 and NCX1: A Novel Strategy for Treating Cancer‐Induced Bone Pain by Modulating Pain Sensitization and Neuronal Inflammation
Source: CNS Neurosci Ther. 2025 Apr 18;31(4):e70389. doi: 10.1111/cns.70389 (PMC12007017; doi:10.1111/cns.70389)
Supplement: Supplementary file 3 — Table S2 [file CNS-31-e70389-s003.pdf]

Table S2. The sequences for primer

Gene	Primer forward (5'~3')	Primer reverse (5'~3')
Slc8a1	AGGTTCTGTTTGCCTTCGTCC	CATCTGCATACTGGTCCTGGGTA
Slc8a2	TGGAGTTCGGCGATGATGAG	CCATTCCCTGAGATGCCTCG
Slc8a3	GCATATGGGGAGCTGGAGTT	GCTTCCTGTCTGTCACTTCTGAT
Scn9a	TGGACTACAACTGTTTATGGGCAA	TGAGTCATTAGCCGAAACAAGG
Scn10a	TCAGAAGAACAGCCATCAAAGTG	CCCTCTTGCCAGTATCTTTATCAG
Scn11a	AAGCTTTGCTGGTGAGAGTAGAGAC	GAGGCATCACATTCACCACAA
Il6	CATAGCTACCTGGAGTACATGAAGAA	GACTCCAGCTTATCTCTTGGTTGA
Il1b	AGGCTCCGAGATGAACAACAAA	GTGCCGTCTTTCATTACACAGGA
Tnf	CCCTCACACTCACAAACCACC	CTTTGAGATCCATGCCGTTG
Gapdh	CCTCGTCCCGTAGACAAAATG	TGAGGTCAATGAAGGGGTCGT
